# The attitudes of infertile couples towards assisted reproductive techniques in Yazd, Iran: A cross sectional study in 2014

**Published:** 2016-12

**Authors:** Seyed Alireza Afshani, Ali Mohammad Abdoli, Mehrieh Hashempour, Maryam Baghbeheshti, Mohammad Zolfaghari

**Affiliations:** 1 *Social Sciences Department, Yazd University, Yazd, Iran.*; 2 *Research and Clinical Centre for Infertility, Shahid Sadoughi University of Medical Sciences, Yazd, Iran.*; 3 *Student Research Committee, Shahid Sadoughi University of Medical Sciences, Yazd, Iran.*

**Keywords:** *Assisted reproductive techniques*, *Attitude*, *In vitro fertilization*, *Iran*

## Abstract

**Background:**

Knowledge about assisted reproductive techniques (ART) and its procedures affect the attitude of infertile people. Making decisions about the use of ART is affected by one's perception and attitude.

**Objective:**

The aim of this cross-sectional study was to determine the attitude of infertile couples toward applying ART, and to investigate its related factors.

**Materials and Methods:**

A randomized cross-sectional study was conducted on 184 infertile couples who had referred to the Research and Clinical Center of Infertility, Yazd, Iran for diagnosis and treatment in June 2014. The data was collected using a two-part questionnaire containing demographic and attitudinal statements. For data analysis, SPSS statistical software and statistical tests of mean differences (t-test), Pearson correlation and analysis of variance were used.

**Results:**

A significant relationship between spouse's attitude (p<0.01), relative's attitude (p<0.01), the applied knowledge of ART (p<0.01), and attitude of infertile couples toward applying the ART was observed; however, there was not any significant relationship between gender and socioeconomic status toward applying ART (p>0.05).

**Conclusion:**

In conclusion, making a decision and accepting ART can be influenced by couple's attitude, their family's attitude and applied knowledge of ART.

## Introduction

Fertility, and having children have always been considered as the blessings from God in the traditional societies and has cultural importance (1). Infertility is the failure of a couple to conceive a baby after trying to do so for at least one full year. From the medical viewpoint, infertility would affect the couple's life, work, health, personality, identity, quality of life, etc. (2, 3). Also, one's emotional status, for example self-esteem and marital satisfaction, can be affected by infertility (4, 5). According to the data compiled by the World Health Organization (WHO) in 2004, there are 187 million infertile couples in developing countries except China. However, a large part of the world including Iran doesn't tend to utilize modern methods of child birth.

On average, about 10-15% of couples have infertility problems in Iran (6). Assisted reproductive technology (ART), such as for instance artificial insemination and surrogacy, is the technology which offers a lot of people the opportunity to have a child (7). In recent years, considerable success has been achieved in the field of assisted reproductive modern technology, while making decisions about it is affected by the people's perceptions and expectations as well as attitudes toward the use of these technologies. A lot of systematic reviews covering ART have been published (8, 9). 

Nowadays, scientists conduct studies that evaluate the familiarity with and also the attitude towards ART and its procedures all over the world (10-13). In this regard, it has been claimed that social factors affect the attitude of infertile people, for example when they want to choose between two ART procedures (14). While the technical, social, ethical and legal issues of ART have been well documented all over the world as well as Asia, a lot of Iranian people don’t know about the exact use of this technology (15-17).

Happiness, well-being, and the need to be loved and give love are the motives for wanting children. According to the high desire of parenthood among young people and their attempts to have children, it is important to know their attitude toward ART in order to help them make the best decisions (18-21). 

The aim of the present study was to determine the attitude of infertile couples toward applying ART, and to investigate its related factors.

## Materials and methods

**Ethical approval**

The research was approved by the Ethics Committee of the Faculty of Social Sciences, University of Yazd on 9^th^ January 2009. All participants agreed to participate in the research voluntarily through written informed consent. Tape recordings were handled confidentially and anonymously by giving participants numbers and all identifying markers were removed.

**Research method**

This randomized cross-sectional study was carried out in June 2014. The sample size, based on the Cochran formula (CI=95%), comprised of 184 subjects including both 86 male and 98 female from totally 300 (statistical population) infertile couples who referred to the Research and Clinical Center for Infertility, Yazd, Iran for diagnosis and treatment in June 2014. The demographic information of the population is completely clarified in the result of this paper. Inclusion criteria were definite infertility diagnosis and intention to apply ART.

**Research** **questionnaire**

A researcher-made questionnaire was applied. The questionnaire consisted of two parts: part 1 contained questions about the demographic characteristics of participants (e.g. gender, education level, education level of spouse, employment status, monthly income, consanguinity with the spouse). While, part 2 contained a researcher-made questionnaire about participants’ attitude toward ART. The questionnaire explores couple’s perspective on ART which were defined as a set of methods of ART including in vitro maturation (IVM), intra-uterine insemination (IUI), intra-cytoplasmic sperm injection (ICSI) and in vitro fertilization (IVF). The answers were noted by the researcher.

The content validity was conducted in the present research. In this case we attempted to choose the items which assay the variables in the study; the items were evaluated by the previous similar researches under the supervision of professional professors. Finally, the last edition of the questionnaire was reviewed by the authorities and the content validity was approved. To ensure the reliability of the questionnaire, the Cronbach's alpha index was determined by the use of pretest data and calculated by SPSS software. The Cronbach's alpha more than 0.7 shows the reliability of the questionnaire. The Cronbach's alpha index was as mentioned below: the attitude toward applying ART (0.70), awareness of ART (0.89), surrounding's people attitude (0.81), family attitude (0.74), spousal attitude (0.71), and religiosity (0.68).

**Awareness and attitude** **definition**

In order to evaluate the awareness of ART among couples, the participants should answer 6 items including: 1) In my family, applying ART makes me feel embarrassed. 2) I don't have enough knowledge about ART. 3) I've heard about ART very much. 4) I have enough knowledge about ART and its process. 5) I have read about ART very much. 6) I know the religious aspects and the exact laws on applying ART. 

The items were assessed from 1-5: completely disagree (1), disagree (2), no opinion (3) agree (4), completely agree (5). The range of variance for awareness of ART was from 6 up to 30, in three groups includes low (6-14), medium (15-22) and high (23-30).

In order to evaluate the attitude toward ART among couples, the participants should answer 9 items including: 1) ART is supposed to give us peace of life. 2) After bearing a child through ART, we will be full of vitality. 3) After bearing a child through ART, a lot of our problems will be resolved. 4) I would feel bad about applying ART. 5) ART process usually fails. 6) I'm sure of the secrecy about the ART project. 7) Applying ART is my last hope. 8) Applying ART makes me feel guilty. 9) Most of couples, who used ART, were not satisfied with that. 

The items were assessed from 1-5: completely disagree (1), disagree (2), no opinion (3) agree (4), completely agree (5). The range of variance for the attitude toward applying ART was from 9 up to 45, in three groups includes low (9-21), medium (22-33) and high (34-45).

**Statistical analysis**

The data was analyzed by SPSS software (Statistical package for the social sciences, version 20 SPSS Inc, Chicago, Illinois, USA) and statistical tests of mean differences (t-test), Pearson correlation and analysis of variance. p˂0.05 were considered significant.

## Results

**Demographic information**

In the present study, 184 candidates were selected as the participants. The average age of the participants was 31±5.003 yr old. There were 86 men (46.7%) and 98 women (53.3%). The level of education was 2 in primary education, 18 junior high school, 86 high school and 78 graduated. In addition, 111 person had a job and 73 were unemployed (Table I) while 76 of their partners had a monthly income of less than 100$.

**Comparing women's attitude to that of men toward ART**

According to t-test results, there was no significant statistical difference (p>0.05) between two sexes regarding their attitude toward ART (Table II).

**Attitude toward ART**

The results of Pearson correlation test (Table III) revealed that the family attitude was highly correlated with spousal attitude (r=0.377, p<0.01), the attitude of surrounding people (r=0.379, p<0.05) and attitude toward ART (r=0.325, p<0.05). Moreover, spousal attitude had a good correlation with surrounding people's attitude (r=0.365, p<0.01), and both of these factors were significantly correlated with attitude toward ART (p<0.01). Also, there was a positive correlation between awareness and attitude toward ART (r=0.311, p<0.01). As mentioned above, the correlation between socioeconomic status and attitude toward ART was not significant (p>0.05). 

**Perspectives on ART according to the awareness**

There was a significant statistical relationship (p<0.01) between the variables including the participant's awareness of ART, his/her partner's awareness of ART, his/her family's viewpoint on ART and their attitude to ART. The majority of participants were aware of ART (Table IV). In other words, as the infertile couple and the people around them were more aware about the ART, their attitude towards new therapeutic methods was more positive.

**Table I:** Demographic characteristics of the participants

**Variables**		**Total (%)**	**Female (%)**	**Male (%)**
Age (years) *		31 ± 5.00	29.30 ± 4.75	32.94 ± 4.56
Educational level **				
	primary education	2 (1.2 %)	1 (0.6%)	1 (0.6%)
	junior high school	18 (9.6%)	5 (2.6%)	13 (7.0%)
	high school	86 (46.7%)	48 (26.1%)	38 (20.6%)
	graduated	78 (42.5%)	44 (24.0%)	34 (18.5%)
Employment status**				
	non-working	73 (39.7%)	68 (37%)	5 (2.7%)
	employee	111 (60.3%)	30 (16.3%)	81 (44.0%)
Place of residence **				
	urban	36 (19.5%)	19 (10.3%)	17 (9.2%)
	rural	148 (80.5%)	79 (43.0%)	69 (37.5%)
Relationship **				
	3^rd- ^degree relative	57 (31.0%)	24 (13.0%)	33 (18.0%)
	4^th^^- ^degree relative	39 (21.2%)	24 (13.0%)	15 (8.2%)
	no kinship	88 (47.8%)	50 (27.3%)	38 (20.5%)

* Data are presented as Mean ± SD.

** Data are presented as n (%).

**Table II:** Male's attitude in comparison with female's attitude towards assisted reproductive technology

**Sex**	**Mean ** **±** ** SD**	**p-value**
Male	37.14 ± 4.58	0.51
Female	36.12 ± 1.72	

Data are presented as the mean ± SD. Evaluated by Independent t-test and p<0.05 considered as significant.

**Table III:** Pearson correlation between attitude and awareness among participants

	**Socioeconomic status**	**Family attitude**	**Spousal attitude**	**Surrounding's attitude**	**Awareness**	**Attitude towards ART**
Socioeconomic status	1					
Family attitude	-0.009	1				
Spouse attitude	0.178	0.377 **	1			
Surrounding's attitude	-0.133	0.379**	0.365**	1		
Awareness	-0.002	0.212**	0.160*	0.055	1	
Attitude towards ART	0.011	0.325**	0.236**	0.282**	0.311**	1

Data are presented as correlation coefficient and evaluated by Pearson correlation test.

* The level of correlation= 0.05

** The level of correlation= 0.01

**Table IV:** Knowledge of participants about assisted reproductive technology and their attitude

**No.**	**Question**	**Mean ± SD**
1	Doing ART can make peace in our family.	4.15 ± 0.80
2	With having a baby by ART methods, our life will have a new meaning.	4.31 ± 0.70
3	With having a baby by ART methods, our life’s problems will be solved.	4.05 ± 0.83
4	I don’t have a good feeling about doing ART.	3.32 ± 0.97
5	ART is successful most of the time.	3.31 ± 0.94
6	I have complete trust that ART remains confidential.	3.92 ± 0.86
7	Doing ART is the only hope we have.	3.08 ± 1.16
8	I feel guilty about doing ART.	3.73 ± 0.94
9	Most of the people, who have done this before, are not satisfied.	3.44 ± 0.84
10	Doing ART is a shame in our family.	3.66 ± 0.96
11	I have a lot of information about ART.	2.99 ± 1.03
12	I heard a lot about ART.	3.35 ± 1.05
13	I have enough information about ART and its next steps.	3.03 ± 1.12
14	I’ve read a lot about ART.	2.93 ± 1.11
15	I have enough information about religious and legal provisions of ART.	3.14 ± 1.10
16	Relatives and neighbors don’t have a good opinion about ART.	3.04 ±1.07
17	Most of our relatives disagree with doing this.	3.35 ± 1.07
18	Our relative's perspective about ART is not tolerable.	3.1 ±1.10
19	Nowadays most people take easy with ART.	3.72 ± 0.99
20	People don’t say good things about ART.	3.03 ± 1.02
21	Most of my spouse relatives disagree with doing this.	3.47 ± 1.12
22	The child that will be born as the result of ART will always feel embarrassed.	4.03 ± 0.86
23	I prefer that our relatives and neighbors do not be informed about this.	2.14 ± 1.17
24	I'm always worried about the tags others put on me.	3.01 ± 1.24
25	My spouse family insists a lot about doing ART.	3.19 ± 1.09
26	My own family encourages me for ART.	3.50 ± 1.10
27	My father doesn't like ART.	3.44 ± 1.01
28	My mother doesn't feel good about ART.	3.52 ± 1.07
29	My brother and sister feel good about ART.	3.58 ± 1.05
30	My father-in-law doesn't like ART.	3.42 ± 0.97
31	My mother-in-law doesn't like ART.	3.47 ± 1.05
32	My brother and sister-in-law feel good about ART.	3.28 ± 1.07
33	My husband is not interested in ART.	3.71 ± 1.16
34	If we don't do this, maybe we get divorce.	3.93 ± 1.20
35	My spouse is doing this, just because of his family.	3.89 ± 1.12
36	My husband and I don't have any problem with doing ART.	3.89 ± 1.04
37	My spouse family don't have good view about ART.	3.58 ± 1.05

Data are presented as the mean ± SD. Evaluated by Descriptive statistics.

## Discussion

The Research and Clinical Center for Infertility was established in Yazd with the attempt of some practitioners in Yazd Shahid Sadoughi University of Medical Sciences in 1989. At present, up to 9000 couples have referred to this center for infertility treatment annually. This center was introduced at the end of 2000 by Educational Deputy of Iranian Ministry of Health and Medical Education as "Academic Pivot for Infertility Education and Research Festival". Yazd Infertility Center is imperious because of doing various studies in different fields such as reproduction and genetics and treating thousands of infertile couples using fertility techniques. Up to now, many research activities were performed in this center including the present study.

This study had shown that there was not any difference between men and women's attitude toward ART. In a general sense, Iranian women show severe emotional reactions against infertility more than Iranian men. They experience more psychological turmoil because of both the social emphasis on the role of being a mother and fear of losing marriage. This situation disturbs women the most and they easily lose their self-esteem; consequently, they look for more information about fertility, start the treatment as soon as possible, and become more hopeful of having a child. Fooladi *et al* showed that the men who suffer from infertility also have the desire to have a baby (22). Men experience infertility differently from women (23). In general, while infertility is a problem for families, it can harm spousal relationships and threaten marriages (24).

Surprisingly, it was found that the socioeconomic status did not have an effect on the couple's attitude. This result is inconsistent with the findings of previous studies. Since the treatment of infertility is costly, and for some of couples, it is necessary to be under treatment for years, so they have to refer to a clinical center to see the specialist several times. Moreover, some couples have to travel to another city which is more costly, as there are no specialists at their city of residence. These issues can affect their attitude and some of them may leave the whole treatment, therefore it can be claimed that socioeconomic status may change the couple's attitude (25-28). But in this study, the majority of clients were from rural areas, and 37.5% of them had an income less than 100$; so, there was not a great difference between their incomes, and there was not a statistical difference between their attitudes.

It is obvious that when couples have a positive attitude toward ART, they can accept it easier. In the current study a significant statistical relationship was observed between the couple's attitude and their family's attitude. Infertility can be stressful for couples; it may have an effect on the quality of their relationship with their family, as well as the relationship between husband and wife. In such a critical situation, the full support of families can influence the couple's mental health. If a couple's family has full understanding and a great empathy with them, they can accept the reality with less difficulty. A study showed that women above 35 years old receive ART better, maybe because they concur with their problem and decide to solve that (29). Therefore, being supported by family reduces the couple's negative thoughts and loneliness, consequently, their positive point of view makes them more willing to choose ART. There are a number of studies on this issue which provide good evidence (30).

Modern technology and advances in ART can be a great help for an infertile person. Even it can be promising to post-menopausal women, as they may wish to experience motherhood and raise children (31). It has been observed that a large number of infertile couples have already benefited from ART in Iran (32). However, making decision for choosing ART as a way of becoming fertile is based on the people's awareness and attitude, as attitude can have an effect on the infertile person to undergo oocyte donation (33, 34). Infertility can make the individuals anxious and depressed, but ART can depress them because they may think the child born by ART is not theirs, but from another one's gamete (35). Some of infertile couples may be fearful of birth defects, as some studies showed that birth defects are higher in infants conceived following ART as compared to spontaneously conceived children (36). So, awareness among infertile couples should be raised to help them make the best decision about getting help from ART.

**Limitations**

Studying a more homogeneous group would allow the researchers to gain in-depth knowledge of the cultural context and influences on attitude. A study which compares the differences in parenting desires across different cultural groups in Iran might be complementary in the future. The results can therefore not be generalized to all people who use ART or to all areas of Iran, although it may be applicable to similar contexts. It is not possible to know if participants in this study were representative of all the opinions about our questions; however, the fact is that the clients might not have understood the exact meaning of our questions, because they were from various cities and had different accents.

**Recommendations**

Despite the limitations, specific recommendations entail this study. It can be applicable to other communities with similar cultural backgrounds.

1. First of all, patients should be referred to a counselor in order to become aware of all costs, risks, procedure and benefits and decide consciously and choose the best method. Also, the counselor should be aware of the whole process of ART and have the ability of convincing them if they refuse to do that due to traditional thinking. 

2. We must assure that the couple is not able to have a child naturally; sometimes couples aren't willing to use ART because they think natural fertilization is a kind of superiority. It should be clarified for them that there is no difference between these two methods of fertilization.

3. Some of the clients were from rural areas with low literacy, so they had less information about ART than educated people. In this case, the role of broadcasting is more prominent. 

4. Sometimes, infertile couples would rather not do this due to the exorbitant costs imposed on them. It is suggested that organizations need to cost more insurance to help infertile couples in order to warm-up the family through fertility assistance, so that no more couples are deprived of this blessing because of infertility costs.

## Conclusion

In conclusion, making a decision and accepting ART can be influenced by couple's attitude, their family's attitude, and their awareness. Infertile couples with the most positive attitude are more willing to choose ART to become parents. Since there are still some people who are unaware of ART, specialists and health professionals should raise their awareness and increase their knowledge in order to help the clients choose the best.
